# How to assure access of essential RMNCH medicines by looking at policy and systems factors: an analysis of countdown to 2015 countries

**DOI:** 10.1186/s12913-018-3766-6

**Published:** 2018-12-07

**Authors:** Jane Briggs, Martha Embrey, Blerta Maliqi, Lisa Hedman, Jennifer Requejo

**Affiliations:** 1 Management Sciences for Health, 4301 N. Fairfax Dr. Suite 400, Arlington, VA 22203 USA; 2 Department of Maternal, Newborn, Childhood and Adolescent Health, World Health Organization, 20, avenue Appia, CH-1211, Geneva 27, Switzerland; 3 Policy, Access and Use Unit, Department of Essential Medicines and Health Products, World Health Organization, 20, avenue Appia, CH-1211, Geneva 27, Switzerland; 4 Johns Hopkins Bloomberg School of Public Health, Baltimore, MD 21205 USA

**Keywords:** Countdown to 2015, Reproductive, Maternal, Newborn, and Child health, Medicines, Health commodities, Access, Pharmaceuticals, Policy, Health systems

## Abstract

**Background:**

In 2000, the Millennium Development Goals set targets for social achievements by 2015 including goals related to maternal and child health, with mixed success. Several initiatives supported these goals including assuring availability of appropriate medicines and commodities to meet health service targets. To reach the new Sustainable Development Goals by 2030, information is needed to address policy and systems factors to improve access to lifesaving commodities.

**Methods:**

We compiled indicator data on 15 commodities related to reproductive, maternal, newborn, and child health (RMNCH) and analyzed them across 75 Countdown to 2015 countries from eight regions to identify problems with specific commodities and determinants of access. The determinants related to policy, regulatory environment, financing, pharmaceutical procurement and supply chain, and information systems. We mapped commodity information from four datasets from the World Health Organization and the United Nation’s Commission on Life Saving Commodities creating a stoplight dashboard to illustrate countries’ environment to assure access. We also developed a dashboard for policy and systems indicators for select countries.

**Results:**

The commodities we identified as having the fewest barriers to access had been in use longer, including oral rehydration solution and oxytocin injection. Looking across the different systems and policy determinants of access, only Zimbabwe had all 15 commodities on both its essential medicines list and in its standard treatment guidelines, and only Cameroon and Zambia had at least one product registered for each commodity. Senegal alone procured all tracer commodities centrally in the previous year, and 70% of responding countries had costed plans for maternal, newborn, and child health. No country reported recent stock-outs of all the 15 commodities at the central level—countries always had some of the 15 commodities available; however, products with frequent stock-outs included misoprostol, calcium gluconate, penicillin injections, ceftriaxone, and amoxicillin dispersible tablets.

**Conclusions:**

This analysis highlights country deficiencies in policies and systems, such as incoherent policy guidelines, problems in product registration, lack of logistics data, and central-level stock-outs that may affect access to essential RMNCH commodities. To tackle these deficiencies, countries need to integrate commodity-related indicators into other health monitoring activities to improve service quality.

**Electronic supplementary material:**

The online version of this article (10.1186/s12913-018-3766-6) contains supplementary material, which is available to authorized users.

## Background

Following the United Nations (UN) Millennium Declaration of 2000, eight Millennium Development Goals (MDGs) were issued to set targets for social achievements by 2015 [1]. MDGs 4 and 5 set goals to reduce under-five child mortality by two-thirds and improve maternal health, including reducing maternal mortality by three-quarters, and achieving universal access to reproductive health.

**Fig. 1:**
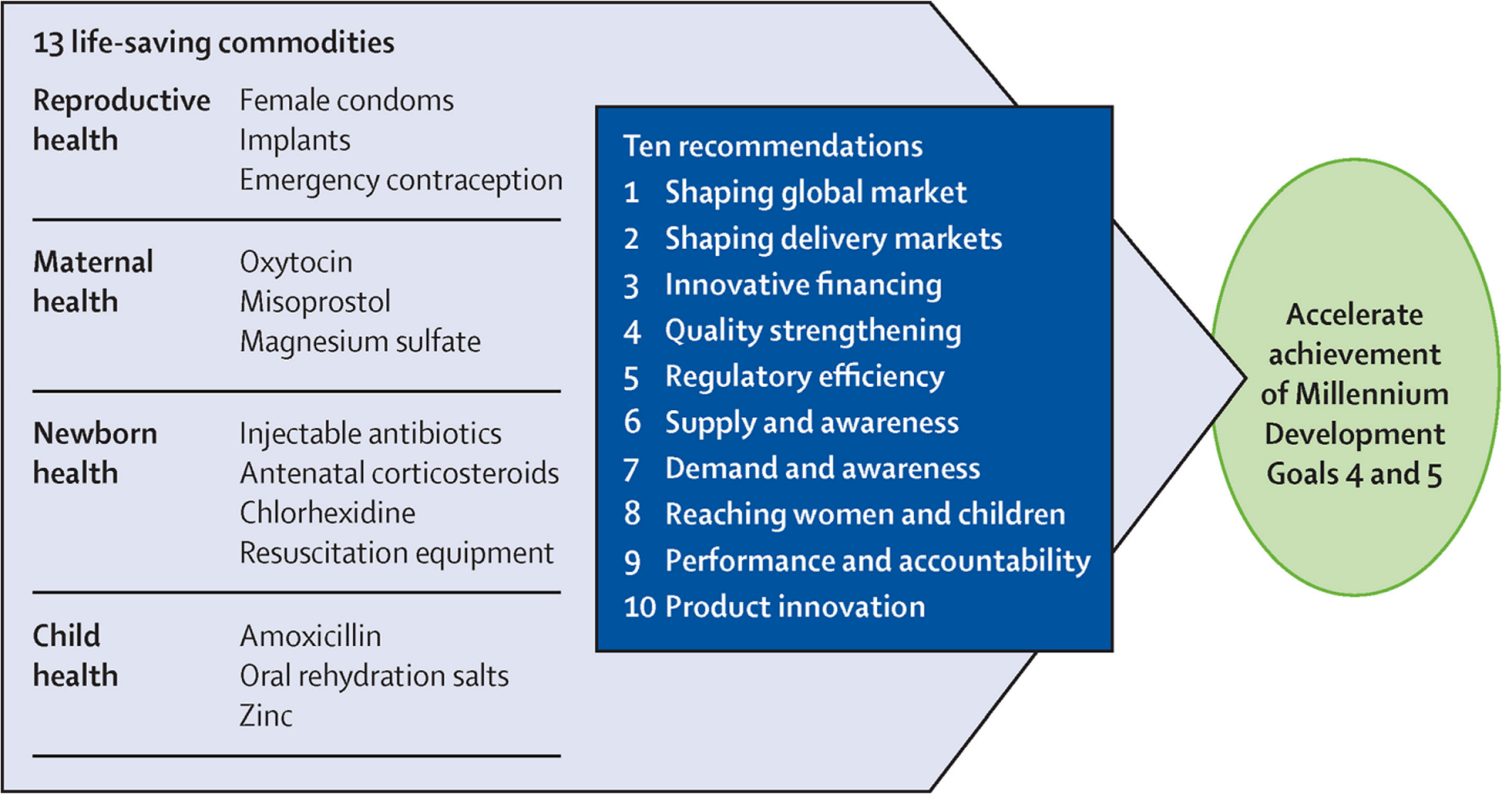
UNCoLSC commodities recommendations and the lifesaving commodities. Source: Pronyk et al. with permission [8]

The MDG agenda for reproductive, maternal, newborn, and child health (RMNCH) was actively supported by the 2010 launch of the UN’s Every Woman Every Child movement. Later in 2012, the UN’s Commission on Life Saving Commodities (UNCoLSC) was initiated in support of Every Woman Every Child and focused on improving access to 13 neglected lifesaving commodities to treat major causes of maternal, newborn, and child deaths, such as postpartum hemorrhage, eclampsia, newborn sepsis, and childhood diarrhea and pneumonia [2]. Under the UNCoLSC, 10 primary actions were identified to address barriers preventing access to these lifesaving commodities [3] (Fig. 1).

By 2015, 50 and 69 countries of the priority 75 countries under the Countdown to 2015 initiative had not met the MDG 4 and 5 targets, respectively [4, 5]. The MDGs were followed by the ambitious Sustainable Development Goals (SDGs) to 2030 [6]. SDG3 focuses on ensuring healthy lives by 2030 and includes sub-goals on maternal, newborn, and child health as follows:

- SDG3.1. By 2030, reduce the global maternal mortality ratio to less than 70 per 100,000 live births
- SDG3.2. By 2030, end preventable deaths of newborns and children under 5 years of age, with all countries aiming to reduce neonatal mortality to at least as low as 12 per 1000 live births and under-five mortality to at least as low as 25 per 1000 live births

To address the new targets, the UN developed a Global Strategy for Women’s, Children’s and Adolescents’ Health (2010; revised for 2016–2020) with an operational plan to support achievement of the SDGs [7]. Progress toward SDG3 depends to a large extent on access to quality medicines and health products for maternal and child health, including the 13 UNCoLSC priority commodities. These commodities are affordable and should be widely available. However, access in many low- and middle-income countries is hampered by systemic problems, such as lack of supportive policies and regulations, insufficient financing and skilled human resources, and weak supply chains [8].

The 13 UNCoLSC commodities were not included in commodity-focused procurement initiatives in the way that HIV commodities are procured through PEPFAR, for example, but rather than creating yet another vertical supply chain, the UNCoLSC approach focused on leveraging existing systems to improve access [9].

Some of the efforts to monitor access to appropriate RMNCH commodities in the MDG era considered policy and system issues, but limited their scope to a certain condition or medicine, for example, postpartum hemorrhage, pre-eclampsia, or zinc for diarrhea [10–12]. While it makes sense to focus on specific areas of health care workforce training and service demand generation, creating separate supply systems for each commodity would clearly be inefficient; in addition, managing one health product at a time rather than managing a system for all health products creates competition for resources. Given that saving the lives of women and children depends on a well-functioning pharmaceutical system, tracking access to essential commodities, especially in high-burden countries, is crucial and should also be included in the process of tracking the SDGs, to which Countdown to 2030 is committed [13]. This will remain critical in the SDG era and in the push for universal health coverage to ensure that the unfinished agenda for women’s and children’s health is not forgotten.

The purpose of this review was to produce an inventory of available data on policy and systems factors affecting access to RMNCH commodities in the 75 Countdown 2015 countries, while acknowledging data limitations. The review contributed to the cross-cutting research conducted by Countdown’s technical working groups and considers the policy and regulatory environment, financing, pharmaceutical procurement, supply chain management, and supportive commodity information systems [10, 14, 15].

We developed our objectives with a view toward providing information for the Countdown to 2030 group to consider as they track country progress. The specific objectives are as follows:

- Compile indicator results related to policy and systems determinants of access to the 13 commodities selected by the UNCoLSC
- Analyze those indicators across the Countdown countries for which data was available
- Determine which commodities have more policy and systems barriers
- Determine which policy and systems factors are more often in place

## Methods

We took available data from two major RMNCH commodity surveys and analyzed them against a set of policy and pharmaceutical system determinants of access. From the results, we identified efforts needed to assure access to these products and improve maternal and child health outcomes. We adapted the 13 UNCoLSC products to use as our tracer list (Table 1). We excluded neonatal resuscitation devices because of our focus on medicines rather than equipment and added calcium gluconate because of its importance as a treatment for accidental overdose of magnesium sulfate; in addition, the injectable antibiotics were disaggregated as gentamicin and ampicillin/benzyl penicillin.

**Table 1:** List of lifesaving commodities included in this review

1. Female condoms	
2. Implants	
3. Emergency contraception	
4. Oxytocin injection 10 IU	
5. Misoprostol tabs 200mcg	
6. Magnesium sulfate injection	
7. Calcium gluconate	
8. Gentamicin injection	
9. Ampicillin or benzyl penicillin injection/procaine pen or alternative	
10. Ceftriaxone	
11. Dexamethasone injection or alternative injectable steroid	
12. Chlorhexidine	
13. Amoxicillin 250 mg dispersible tablets (DT) or syrup	
14. Oral rehydration solution (ORS) sachets	
15. Zinc dispersible tablets	

### Choice of indicators

A comprehensive list of indicators to predict sustainable access to medicines or the readiness of pharmaceutical systems to deliver lifesaving commodities does not exist. For example, while in some countries, ORS has favorable regulations, procurement policies, and cost, its use remains low due to factors such as caretaker bias or lack of knowledge [16]. Access to emergency contraception, on the other hand, may be affected by policies on prescription versus over-the-counter distribution status.

Although a study of the supply chain and related performance indicators to monitor the availability of the 13 lifesaving commodities was conducted, no methods exist for tracking the systems and policy factors related to commodity access [17]. We argue that products can be available on the market, and yet many women and children are still not benefiting from their lifesaving properties; for example, a country may have approved the use of ORS and amoxicillin, but has no policy supporting community case management of diarrhea or pneumonia. Additional national policies, such as those related to commodity selection and procurement and pharmaceutical management support systems such as robust logistics management information also play vital roles in assuring access to lifesaving medicines. In developing our set of indicators (Table 2), we take a holistic approach that looks beyond just product availability to include key policy and systems determinants that affect the patient’s ability to access both products and services by focusing on policy, regulatory systems, procurement, and financing, as well as supply chain management and information systems [18]. The choice of indicators was also influenced by the availability of data in the four data sources used [8]. We also included financial indicators that can affect access to commodities; for example, having a costed RMNCH plan can facilitate resource mobilization for commodity procurement, while patient fees for medicines or services can be a barrier to access [19, 20]. Eight indicators are commodity specific. Additional file 1 includes a more detailed explanation of the access framework and a summary of the evidence supporting our indicators.

**Table 2:** Indicators of policy and pharmaceutical systems used for the review

Policy	
1. % of countries with a current (updated in the last 2 years) essential medicines list (EML)	
2. % of countries with an EML that includes tracer RMNCH medicines^a^	
3. % of countries with RMNCH tracer medicines in standard treatment guidelines (STGs)	
4. % of countries where RMNCH tracer medicines in the STG are also on the EML^a^	
5. % of countries with a national policy on the use of community management of pneumonia	
6. % of countries with a national policy on the use of community management of diarrhea	
Regulatory	
7. % of countries where tracer medicines have at least one product registered for use in country^a^	
8. % of countries where quality problems are reported	
9. % of countries where medicines (including RMNCH medicines) products are routinely sampled for quality testing	
Procurement	
10. % of countries where RMNCH commodities are procured centrally ^a^	
Financing	
11. % of countries with a costed MNCH plan	
12. % of countries with fees for services in the public sector	
13. % of countries with fees for services where women and children under 5 are exempt from paying for RMNCH services or medicines	
14. % of countries with a policy to provide RMNCH commodities free of charge in public sector ^a^	
Supply Chain Management	
15. % of countries with a pull (demand-based) distribution method to health facilities	
16. % of countries with stock outs of RMNCH tracer products reported at CMS in last 3 years^a^	
Information systems	
17. % of countries with a logistics management information system (LMIS) to track stock-level/consumption of medicines (paper, electronic, or mobile)	
18. % of countries where all tracer RMNCH commodities are included in LMIS^a^	

^a^Indicator reported for each commodity

### Data sources

We extracted data for the selected indicators on policy, regulatory, procurement, financing, and information systems from four relevant data sources:

- World Health Organization (WHO) health systems and policy survey dataset collected from December 2013 to June 2014. This data was collected every other year on the 75 Countdown to 2015 countries [5]. The data comes from surveys of government authorities that are administered and compiled by WHO headquarters staff and coordinated by staff in WHO country offices.
- Regulatory and procurement survey conducted between August 2013 and March 2014 by the WHO Essential Medicines and Health Products Department [21]. This survey used two modules from the *RMNCH Landscape Synthesis* (a situation analysis) from the UNCoLSC. The survey was sent to ministry of health counterparts via the WHO country office in 49 Every Woman Every Child countries [22]. WHO staff in country consolidated the responses and sent them back to WHO headquarters. Responses were received from 22 countries.
- The UNCoLSC had conducted an *RMNCH Landscape Synthesis* in 21 countries by the end of 2016 (http://www.lifesavingcommodities.org/wp-content/uploads/2016/12/Landscape-Synthesis-Overview-2-pager-2016-09-30.pdf). We used this data only if data points were missing from the other two sources—data from 17 of our target countries was available at the time of this analysis.
- To complement information on essential medicines lists not included in the previous data sources, we searched the WHO Essential Medicines and Health Products Information Portal to identify uploaded copies of national EMLs (http://apps.who.int/medicinedocs/en/).

For some indicators, we had to combine data from different data sources; for example, not all the commodities in our tracer list were included in the WHO health systems and policy survey, so we used data from the regulatory and procurement survey to fill in. If country responses between the different data sources did not align, we used the most recent data. Additional file 2 shows which data sources were used for which indicator.

### Data analysis

We consolidated data and calculated the percentages of countries reporting on each indicator. We used specific indicators to determine how well each tracer item was favorably supported by the policy, regulatory, procurement, and financing systems and to highlight gaps in support. We attempted to map all existing information from the data sources in relation to commodities for all 75 Countdown countries, however, some indicators had many fewer data points depending on what information was available. Given this variability in the amount of data per indicator, including some with very few data points, we did not calculate statistical correlations. The analysis, therefore, presents a snapshot of the situation related to pharmaceutical systems and policy factors across a sample of Countdown countries.

We set up a stoplight dashboard of these determinants of access to easily illustrate the degree to which countries have a favorable policy environment. We determined the performance cut-off levels for the colors based on other stoplight dashboard examples and by consulting with subject area experts: green’s threshold of 75% and over implies stronger system performance; yellow (60–75%) shows that the system required further strengthening; and under 60% (red) highlights weaknesses or deficiencies that require attention. The three bands illustrate differences, but can also be used to prioritize action and track performance. The dashboard color does not reflect the amount of data available for each indicator (i.e., number of Countdown countries responding). We felt that three bands were easier to interpret and use for tracking and prioritizing action than a more complex visualization using four or five bands as used in other analyses [8, 23, 24].

We averaged the mean percentages of the indicators for individual commodities and policy/system factors to show which commodities and policy/system factors were consistently associated with more problems.

## Results

**Fig. 2:**
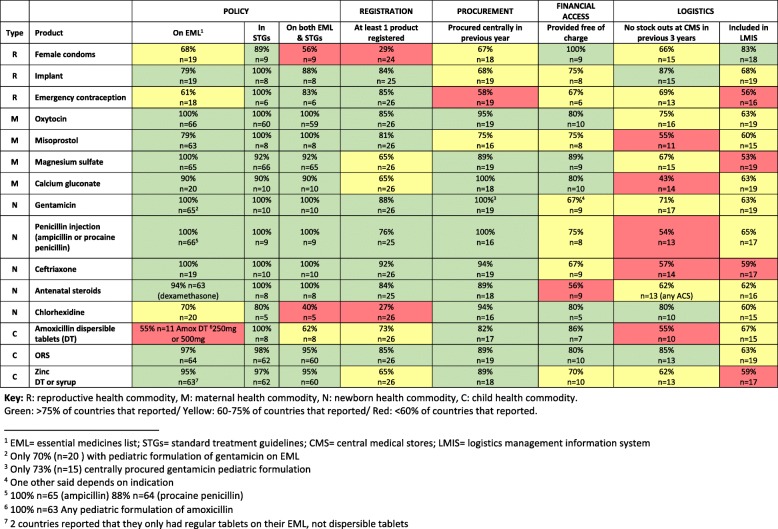
Dashboard of results for 8 policy and system indicators for each of the 15 tracer commodities (n shows # of countries responding)

The stoplight dashboard (Fig. 2) presents all 15 commodities and the performance of the eight commodity-specific indicators from the list in Table 2. The following sections discuss the areas that require strengthening across the eight commodity-specific indicators or across commodities.

Using the averages of the means in Fig. 2, Table 3 shows the consolidated score for each commodity in descending order. Commodities with the fewest problems included ORS, oxytocin, and gentamicin and penicillin injections, and those associated with more policy and systems problems included amoxicillin dispersible tablets, emergency contraception, female condoms, and chlorhexidine. Table 4 shows which policy and system factors were most challenging—registration of commodities, tracking of commodities by LMIS, and lack of stock-outs at the central level, while fewer problems across the commodity set were noted with policy indicators such as inclusion in the EML and STGs.

**Table 3:** Average percentages for 15 RMNCH lifesaving commodities across the 8 policy and system indicators

Commodity Type	Commodity	Average % (in descending order) ^a^
Child	Oral rehydration solution	87
Maternal	Oxytocin	87
Newborn	Gentamicin	86
Newborn	Penicillin injection	84
Newborn	Ceftriaxone	84
Reproductive	Implant	81
Maternal	Magnesium sulfate	81
Newborn	Antenatal steroids	81
Child	Zinc	79
Maternal	Calcium gluconate	78
Maternal	Misoprostol	78
Child	Amoxicillin dispersible tablets	73
Reproductive	Emergency contraception	72
Reproductive	Female condoms	70
Newborn	Chlorhexidine	66

^a^Means of the average percentages presented in Fig. 2 came from different sample sizes and were rounded to whole numbers

**Table 4:** Average percentages for 8 policy and system indicators for 15 RMNCH lifesaving commodities

Policy system factor	Average % in descending order^a^
In standard treatment guidelines	96
On essential medicines list	86
On both essential medicines list and standard treatment guidelines	86
Procured centrally in previous year	86
Policy to provide free of charge to patients	76
At least one product registered by the national drug authority	72
No stock-outs at central medical store in previous 3 years	66
Tracked by logistics management information system	63

^a^Means of the average percentages presented in Fig. 2 came from different sample sizes and were rounded to whole numbers

We also developed a dashboard for the whole set of policy and systems indicators for a subset of countries as shown in Additional files 3 and 4. For those 10 countries (coded for anonymity) with the most data points available, we mapped systems and commodity indicators to identify bottlenecks to access and their potential relationships to health outcomes—maternal and child mortality.

The results for each policy and system indicator domain presented in Table 4 follow.

### Policy

The WHO defines essential medicines as those that satisfy a population’s priority health needs and therefore should be available at all times. For a country’s essential medicine list to be effective, it has to be maintained regularly. The process for updating an EML includes consideration of disease prevalence, public health relevance, evidence of clinical efficacy and safety, and comparative costs and cost-effectiveness of treatment. An updated EML can be a significant country investment; therefore, the WHO updates its model EML every 2 years to provide guidance. Like the EML, STGs need to be updated to reflect new treatment recommendations as disease patterns and treatment options change. STG revisions are also a significant country investment; evidence must be duly considered by appropriate experts and the guidelines published and widely disseminated. STGs form the basis of health worker training in appropriate treatment; therefore, medicines that are included in the STGs will be in greater demand.

#### Essential medicines lists

Based on the WHO Essential Medicines and Health Products Information Portal, 63 of the 75 Countdown countries had submitted their EMLs, and of those, only 14% had been updated in the previous 2 years (2013 or 2014). While this evidence suggests possible delays in updating EMLs, reporting is voluntary, so information may not be sufficiently complete. The year of EML revision was not collected in the other surveys.

Of the 66 countries that responded to either of the WHO surveys on which commodities were on the national EML, only seven (Democratic Republic of Congo [DRC], Guinea, Kenya, Rwanda, Senegal, Zambia, and Zimbabwe) had all the 15 RMNCH commodities on their current EML; Bangladesh, Cameroon, and Mali had 14 of the 15 RMNCH commodities; and Ethiopia, Malawi, and Uganda included 13 of the 15 commodities. The products that were least likely to be included on the national EMLs were female condoms (68% of responding countries), emergency contraception (61%), chlorhexidine (70%), and amoxicillin DT (55%) (Fig. 2). Some countries may consider the female condom as a medical device and not include it on an EML, while others may not specify condoms by gender on the EML. Emergency contraception (levonorgestrel), chlorhexidine, and amoxicillin may appear on national EMLs, but not in the appropriate dosage form or formulation for RMNCH usage. None of the data sources included information on process or background for why a particular product or dosage form was included in the EML.

#### Standard treatment guidelines

According to the two WHO surveys, 66 of 68 countries reported having at least one of the tracer RMNCH commodities in their STGs; only Zimbabwe included all 15 RMNCH tracer commodities in their STGs. Ethiopia, Guinea, Sierra Leone, and Uganda included all 14 of the commodities they reported on, and Senegal and Tanzania had all 12 that they reported on. Malawi had 12 of the 14 commodities that they reported in their STGs, excluding female condoms and chlorhexidine. The other countries only responded for a few commodities (average of four) but of those few, they had included the majority in their STGs.

#### Alignment of EMLs and STGs

Minimizing the lag time in aligning EML and STG updates—that is, assuring that a commodity is included on both—is important to avoid confusion in procurement and training. We were able to analyze EML/STG alignment in 65 countries, although the number of commodities with responses for each variable differed from country to country; for instance, some responded for only two commodities and others for the full set of tracer RMNCH commodities. Only Zimbabwe had all 15 RMNCH tracer commodities on both the EML and in the STGs; of the remaining 64 countries, 49 (77%) reported on a varying subset of tracer commodities included in the two references and 15 (23%) had a mismatch for one or more commodities between the two policy documents. In this analysis, we did not specify whether the commodity was missing from the STG or the EML, just that the two did not match. Figure 2 showed that commodities for which there was no alignment in the policy documents had been recommended for RMNCH conditions more recently, such as chlorhexidine 7% and amoxicillin DT. We did not consider the publication years of the EML and the STG, so we could not assess if the mismatch was valid—for example, more than 2 years’ lag.

#### Community health worker policy

Using community health workers to dispense amoxicillin, ORS, and zinc improves access to treatment for pediatric pneumonia and diarrhea, and is recommended by WHO and UNICEF [25]. Of 65 responding countries, 88% had a national policy on community case management of diarrhea; whereas, 63% of 63 responding countries claimed to have a national policy permitting community case management of pneumonia. These were tracked separately, because as shown, the uptake of community case management of pneumonia has been slower due to the resistance to using antibiotics at community level. While the national policy does not necessarily give prescribing authority to community health workers, community case management can only be implemented within a permissive policy environment.

### Regulatory

#### Registration

National medicines regulatory authorities (NMRAs) ensure that medicines and health products that circulate on national markets are safe, effective, and meet quality standards. A product should be registered with the NMRA in any country where it is being distributed. Product registration is specific to the manufacturer, the product presentation, and the manufacturing site.

Of the 26 countries for which there were data on registration of one or more products from both the WHO regulatory procurement surveys and the RMNCH landscape synthesis (data for nine countries: Bangladesh, Cameroon, DRC, Ghana, Mali, Mozambique, Senegal, Tanzania, Zambia), only Cameroon and Zambia had at least one registered product for each of the 15 RMNCH commodities. Eleven of the 26 countries had more than 11 of the 15 commodities registered. Analyzing product registration across the countries for each commodity, we found that the products less likely to be registered were female condoms, magnesium sulfate, calcium gluconate, chlorhexidine 7.1%, amoxicillin DT, and zinc tablets or syrup, as shown in Fig. 2. Lack of registration may be understandable for relatively new products such as zinc or new dosage forms such as chlorhexidine for umbilical cord use (7 of 26 countries reporting). On the other hand, more established products such as magnesium sulfate and calcium carbonate were each registered in only 65% of the 26 countries reporting. In five of 26 countries, the registration of several RMNCH commodities including magnesium sulfate had expired. A key finding from the WHO regulatory and procurement survey was the unusual demand and market imbalance for ceftriaxone; for example, there were almost 300 products registered for ceftriaxone across the surveyed countries compared to only 16 oxytocin products. While the registration of several different products for one medicine ensures market competition, 300 registered products are excessive and inefficient.

#### Postmarketing surveillance

Once NMRAs have authorized products to be distributed in a national market, they are then responsible for monitoring product quality and safe use. Of the countries responding to the WHO survey, 77% of 22 countries stated that they have a postmarketing surveillance system that includes sampling and laboratory testing of products as part of a postmarket quality assurance program. Survey questions on reporting of adverse reactions were only included in later versions of the RMNCH landscape synthesis; however, almost all of the 13 countries responding to this question indicated that they had a pharmacovigilance system for adverse event reporting. The question did not include any indication of system functionality—just that it was in place.

### Procurement

Central procurement agencies (and their subordinate agencies) are an important source of data on health products that countries are procuring. The government’s central medical stores (CMS) purchase on behalf of many public health programs and consolidate regional or provincial demand. This allows volume pricing, long-term supply planning, and overall commodity security [26]. Some countries rely on a single CMS, while in others, the CMS decentralizes procurement to subordinate agencies, such as districts or states. The presence of a medicine or health product in a CMS system is an indicator that the medicine is available in the country, at least at the central level.

In the WHO regulatory and procurement survey and the UNCoLSC landscape synthesis, up to 19 countries responded to a question on whether there was centralized procurement for each of the tracer commodities, although not every country responded for every commodity. Senegal was the only country reporting centralized procurement of all 15 RMNCH tracer commodities in the previous year, and four other countries, Bangladesh, DRC, Guinea, and Zambia, centralized their procurement of all the 14 tracer commodities they reported on. Three countries, Malawi, Mali, and Zimbabwe, centrally procured 14 of 15 tracer commodities that they reported in the previous year. Overall, 63% of the countries (12) reported centralized procurements for more than 11 commodities (or 73%) of the tracer 15 commodities in the previous year.

Commodities that were less likely to be procured centrally, as shown in Fig. 2, include misoprostol and the reproductive health commodities, including female condoms, implants, and emergency contraception. All other commodities had been included in a national procurement tender in the previous 12 months in 80% of countries that responded, which varied between 16 and 19 countries for the different commodities. Interestingly, while all 19 countries responded that they procured gentamicin injection centrally, only 73% of 15 countries responded as to whether the pediatric formulation of gentamicin was included in their tender. Two of the countries had not included pediatric formulations of gentamicin on their EMLs.

### Financing

#### Costed plans

A country may finance the procurement of health commodities through government allocations, donor contributions, cost-sharing mechanisms, or combinations of those. We assumed that a costed plan would include resources for procurement of RMNCH commodities and therefore make mobilizing resources easier. Of the countries that responded in the WHO health systems and policy survey as to whether they had costed maternal, newborn, and child health plans, 83% had a costed maternal health plan (of 64 responding), 80% a costed newborn plan (of 61 responding), and 71% had a costed child health plan (of 59 responding). Of the 57 countries that responded to all three questions, 70% had developed all three costed plans. We assume but did not verify that RMNCH commodities were included in the costed plan.

#### User fees for commodities

Additionally, we looked at whether the country was taking steps toward universal health coverage by reducing patients’ economic burden, such as putting a policy in place to provide RMNCH commodities at no charge. Reponses regarding user fees may reflect differences of interpretation, for example, as to whether there was a consultation fee, a fee for medicines, or both. Looking at the economic burden on patients for RMNCH services, 46 of 65 countries (71%) reported in the WHO health systems and policy survey having a user fee policy in the public sector; however, of those 46 countries, 57% reported that newborns were exempt, 59% reported that children were exempt, and 63% of 43 responding countries stated that women were exempt from paying for RMNCH services or medicines.

While more than half of the countries had exemptions for RMNCH services, this does not necessarily assure universal access to RMNCH commodities. Data on whether specific commodities were provided free of charge came from the WHO regulatory and procurement survey and is shown in Fig. 2. Responses came from between 5 and 10 countries depending on the commodity. Of the commodities whose sole indication was for RMNCH, most of the countries responding in the regulatory and procurement survey reported that at least some of the commodities were provided free of charge (Fig. 2); for example, oxytocin is free in 8 of 10 reporting countries, and female condoms are provided at no cost in all nine reporting countries. However, commodities with wider indications than just RMNCH (i.e., antibiotics and steroids) were not provided free of charge as often; for example, gentamicin and ceftriaxone were reportedly provided for free in six of nine countries and steroids in only five of nine countries. Only Zimbabwe offered all 15 RMNCH commodities at no cost; five other countries (Ethiopia, Malawi, Sierra Leone, Tanzania, and Uganda) reported that all the commodities that they reported on were provided free of charge; and for Afghanistan, 93% of the commodities they reported on were provided free of charge. For this analysis, we only looked at the policy in place and not the actual practices on the ground.

### Supply chain management

One would assume that if the health system can ensure consistent central-level availability of commodities, then peripheral facilities are more likely to be stocked. While no country reported recent (within the previous year) stock-outs at the CMS for all 15 commodities in the WHO regulatory and procurement survey and the RMNCH landscape synthesis, 15 of 18 countries (83%) reported a recent stock-out of at least one RMNCH commodity at the central level, and three countries, Cameroon, Ghana, and Guinea, reported recent central level stock-outs of nine or more RMNCH tracer commodities. Commodities that were particularly affected by central-level stock outs as shown in Fig. 2 were misoprostol, calcium gluconate, penicillin injections, ceftriaxone, and amoxicillin DT. Between 10 and 14 countries reported stock-outs at the central level for these commodities (depending on the commodity), of which only around half reported no stock-outs for each of the five commodities. Over 80% of the countries reported no central-level stock-outs of only three products (implants, chlorhexidine, and ORS).

Product availability at facilities is also affected by the distribution mechanism. In a “pull” system, health facilities determine types and quantities of products they need and place orders to the regional or central stores (depending on the system), while in a “push” system, a supplier sends health facilities a predetermined selection and quantity of products in a kit at a scheduled time. Pull distribution is typically more efficient because the orders are linked to consumption at the individual facilities, and so less is wasted [27]. However, the success of a pull system relies on how well the facility staff can manage medicines and calculate their needs. Although countries should aim to implement an order-based distribution system, only 3 of 13 countries responding to the WHO regulatory and procurement survey reported having a pull distribution in place.

### Information systems

A good pharmaceutical logistics management information system provides the necessary data to make sound decisions at all levels of the pharmaceutical system. Eight countries of only 10 that responded to the general question in the WHO regulatory and procurement survey said they had an LMIS, either paper-based or electronic. An LMIS should include RMNCH commodities; however, this presented a challenge. While no country reported all 15 RMNCH commodities in its LMIS, 12 of 19 countries stated that over half of the reported lifesaving commodities were tracked in the LMIS. Interestingly, 19 countries responded to the questions on LMIS related to specific commodities; this could mean they were referring to commodity-specific or program-specific logistics systems or simply that more responded to those specific questions. As shown in Fig. 2, the one commodity most likely to be included in the LMIS was female condoms (83% of responding countries). Those commodities most often left out of the LMIS were emergency contraception, magnesium sulfate, ceftriaxone, and zinc.

### System-wide analysis

In addition to analyzing the system and policy determinants of access across all countries, for the 10 countries with the most data points available, we mapped systems-related and RMNCH-specific commodity indicators (from Table 2) to identify bottlenecks to access and their potential relationship to maternal and child mortality. Given small sample sizes, we were unable to conduct a robust statistical analysis, but the findings are available in Additional files 3 and 4. While more “red lights” on the dashboard are not the only factors contributing to gaps in reductions in maternal or child mortality, addressing weak areas may make a substantive difference for countries that still have significant gaps.

## Discussion

Despite the importance of commodities in effective programming, only one study related to the availability of the 13 lifesaving commodities has been conducted, and its main focus was on supply chain with some additional cross-cutting indicators [17]. There is still no global consensus on a set of standard indicators to monitor systems and policy factors related to commodity access. The indicators that we chose for this review are not the only ones for measuring policy and pharmaceutical system factors that determine access to commodities, but represent key indicators that were readily available through four recent data sources.

Not surprisingly, our analysis showed that the commodities with the fewest problems were generally those that had been in use longer (e.g., ORS, oxytocin, and penicillin and gentamicin injections). Looking at the systems and policy factors across all commodities, the areas that scored lowest were product registration, tracking of commodities by LMIS, and stock-outs at the central level. These weakest points need to be addressed to improve access to commodities in the Countdown countries, including introducing them to the market successfully.

### Essential medicine lists and standard treatment guidelines

Important commodity-related policy issues include but are not limited to the selection of essential medicines and the implementation of STGs that form the basis of training, monitoring, and supervision of health workers to assure appropriate case management, as described in the indicator rationale paper (Additional file 1).

Countries should consider global recommendations to ensure safe and effective treatment, such as use of chlorhexidine 7.1% for cord care in countries with high-neonatal mortality risk and amoxicillin DT 250 mg for pneumonia, when they are revising their EMLs. In addition, because EMLs are often used to make procurement decisions, updates need to include all necessary dosage forms [15, 26]. Some countries with decentralized health systems may also need to assure that new commodities are included on sub-national EMLs as well as the national EML. Harmonizing the processes for updating EMLs and STGs will reduce lag time between them. An expedited EML review process could also reduce lag time if a product has been added to a treatment guideline.

### Registration

The NMRA gives market authorization or “registration” to medicines and health products after reviewing data submitted by the manufacturer, inspecting manufacturing facilities, or assuring that the product has been pre-qualified by WHO, for example. These agencies oversee the country’s pharmaceutical quality assurance system, which is crucial to assure product safety and efficacy; therefore, a key focus of improving access to medicines should be to strengthen product registration systems and assure that commodities are included in a regular postmarketing quality surveillance program to monitor appropriate use, adverse events, and other safety issues. Careful attention should be paid to existing products to ensure that registration status gets renewed prior to expiry.

### Procurement

Centralized procurement often facilitates commodity security and cost-effectiveness due to the economies of scale realized from pooling procurement nationwide. However as shown in Fig. 2, even if commodities are procured centrally, central-level stock-outs still occur due to myriad factors, such as poor quantification or supply planning or weaknesses in inventory management, and although central-level availability clearly affects downstream product availability, it is not the sole determinant. Typically, RMNCH commodities are not managed vertically with dedicated program support, but are included with other national essential medicines, which highlights the importance of strengthening the pharmaceutical system as a whole.

### Financing

Developing a costed plan is an important strategy for a country to mobilize resources to procure commodities from a variety of sources, including domestic funds. This is increasingly important for countries preparing their RMNCH investment case to receive funds through the Global Financing Facility [28]. Policy makers need to carefully consider user fees to reduce barriers to access to RMNCH commodities—particularly in the context of universal health coverage. Additionally, the WHO survey noted that commodities provided free of charge were equally likely to experience stock-outs as commodities supplied at a fee, so this funding strategy does not necessarily increase access.

### Information systems

While an LMIS is not the sole determinant of access, good quality information is critical to quantifying how much of a product should be procured and knowing the stock status at all levels of the health system. Our results showed that RMNCH commodities were not systematically included in an LMIS, which presents challenges to monitoring their availability and accurately forecasting their needs. The costs of setting up a nationwide electronic LMIS and associated dashboards for visibility and decision making can be enormous, but their value in helping improve availability of medicines cannot be underestimated. Countries should seek opportunities to align donor commitments and streamline interventions to improve the use of quality logistics data for decision-making and improved availability of medicines.

### System-wide analysis

The exercise of analyzing the systems and policy indicators for commodities across 10 countries (Additional files 3 and 4) illustrates that most countries had problems with commodity policy and systems issues and still had gaps in achieving the MDG targets. Addressing these identified weaknesses could help close the gaps and reduce maternal and child mortality. If countries analyze these factors for themselves, it could help prioritize their appropriate actions. While this analysis was conducted in the context of the MDGs, the issues clearly apply to the SDG targets as well.

Countries should have a system for ongoing monitoring of policy and systems indicators related to commodities as part of their overall monitoring and evaluation plan to show progress toward the SDGs. Tracking these indicators annually would not be too onerous because the data comes from existing sources. A situational analysis of these determinants of access for commodities could be an important contribution to a country’s annual planning and grant proposal processes, including the development of investment cases under the Global Financing Facility.

### Limitations

This analysis was intended to cover the 75 Countdown to 2015 countries where more than 95% of all maternal and child deaths occurred; however, we did not have data for all countries and across all indicators despite using four different sources. Therefore, we focused on a subset of the 75 countries for which data were available. While the information shown was the most recent at the time we collected it, factors change frequently in countries, so it may no longer reflect the current situation.

Both of the WHO surveys are administered electronically and contain a number of qualitative questions. Responses to these questions depend on the ability of respondents to use the guidance correctly, so respondents may have interpreted questions differently. For example, in the WHO regulatory procurement survey, some may have thought that an LMIS had to be electronic to answer yes—although a functioning paper-based system was also intended; whereas, the RMNCH landscape synthesis did focus exclusively on an electronic LMIS, which presents another limitation to our analysis—comparability between survey tools.

Lastly, the analysis presents a one-time cross-section of results that requires periodic updates to identify and understand trends. We recommend that future research focus on applying the indicators in a standardized way across several countries to validate their usefulness within and across country contexts.

## Conclusions

The RMNCH lifesaving commodities are essential to meeting the SDG targets and improving the health of women and children. Monitoring the policy and systems factors that affect access to these commodities and other essential medicines is critical to understanding a country’s ability to provide them—however—these factors are often forgotten.

This analysis highlights country deficiencies in policies and systems affecting access to essential RMNCH commodities, such as incoherent policy guidelines, problems with product registration, lack of logistics data, weak systems to monitor quality, as well as central-level stock-outs. Insufficient awareness of these indicators and related data make it difficult to monitor progress meaningfully. Monitoring should not just be at a global level, but also at country level to support planning and efficient resource allocation [29]. Countries need to adopt indicators such as these and integrate them into other SDG and RMNCH monitoring activities. The highest-burden countries, particularly, should link these metrics to strategies to improve the quality of available RMNCH services. Additionally, such monitoring supports recommended efforts to increase global and national accountability related to reducing maternal and child mortality [30]. Creating reliable pharmaceutical systems is essential to achieving SDG3, and it will require taking a holistic look beyond just product availability and logistics and toward strengthening other system components that affect access to both products and services [31–33].

## Additional files

Additional file 1: Measuring determinants of access to lifesaving commodities—not just availability. (PDF 541 kb)

Additional file 2: Indicators and data sources. (PDF 70 kb)

Additional file 3: Reproductive and maternal health commodity policy and systems indicators for a subset of countries (countries arranged in order of increasing gap with CD 2015 targets). (PDF 94 kb)

Additional file 4: Newborn and child health commodity policy and systems indicators for a subset of countries (countries arranged in order of increasing gap with CD 2015 targets). (PDF 97 kb)

## Abbreviations

CMS: Central medical stores
DRC: Democratic Republic of Congo
DT: Dispersible tablets
EML: Essential medicines list
LMIS: Logistics management information system
MDGs: Millennium Development Goals
NMRA: National medicines regulatory authority
ORS: Oral rehydration solution
PEPFAR: US President’s Emergency Plan for AIDS Relief
RMNCH: Reproductive, maternal, newborn, and child health
SDGs: Sustainable Development Goals
SIAPS: Systems for Improved Access to Pharmaceuticals and Services [program]
STGs: Standard treatment guidelines
UNCoLSC: United Nations Commission on Life Saving Commodities
UNICEF: United Nations Children’s Fund
USAID: US Agency for International Development
WHO: World Health Organization
