# Supplementary material for: How to assure access of essential RMNCH medicines by looking at policy and systems factors: an analysis of countdown to 2015 countries
Source: BMC Health Serv Res. 2018 Dec 7;18:952. doi: 10.1186/s12913-018-3766-6 (PMC6286577; doi:10.1186/s12913-018-3766-6)
Supplement: Supplementary file 1 — Measuring determinants of access to lifesaving commodities—not just availability. (PDF 541 kb) [file 12913_2018_3766_MOESM1_ESM.pdf]

Measuring Determinants of Access to Lifesaving Commodities—Not Just Availability

Authors: Jane Briggs, Martha Embrey, Beth Yeager

USAID's Systems for Improved Access to Pharmaceuticals and Services (SIAPS) program implemented by Management Sciences for Health (MSH)/

BACKGROUND

A few years ago, a United Nations Millennium Project task force concluded that global programs cannot successfully address individual diseases until more resources are devoted to strengthening entire health systems and that the effectiveness of a health system can be measured by the consistent availability of medicines.[1] The last decade's major global health initiatives, such as the President's Emergency Plan for AIDS Relief and the Global Fund, have helped reduce pharmaceutical prices and supply them to countries in need, but these activities do not automatically lead to *access* to medicines. Progress toward MDGs 4 and 5 depended on the availability of quality commodities essential for restoring and maintaining health of women and children and this will be more important in the Sustainable Development Goal era. In general, these products are affordable and available on the market, and yet many women and children are still not benefiting from their lifesaving properties. We must take a holistic approach that looks beyond product availability to include other components that affect the patient's ability to access both products and services. Figure 1 illustrates one of several medicines frameworks showing that access is more than availability. [2,3,4]

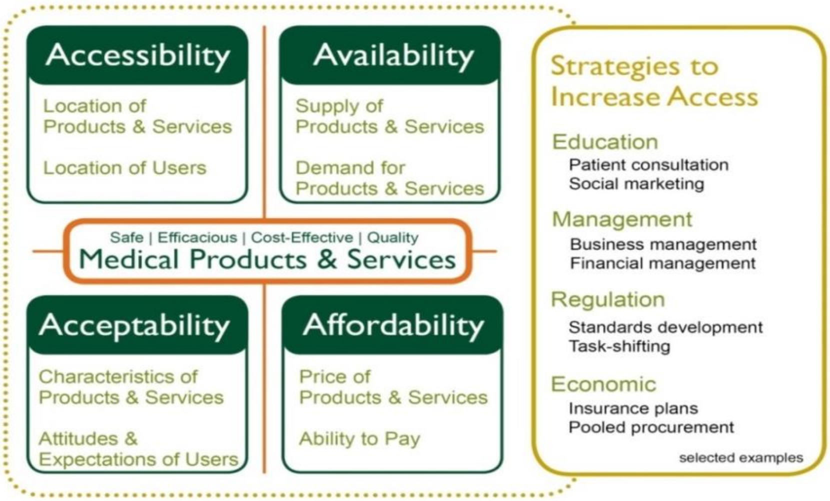

Figure 1. Increasing Access to Medicines Framework. At the core of access is the need to provide medicines and services that are safe, efficacious, cost effective, and of high quality. Often people assume that if medicines are in stock, then positive outcomes will naturally result. But availability is only one aspect of ensuring access to medicines—equally important are accessibility (geography and convenience), affordability (price and ability to pay), and acceptability (cultural and personal preferences).[1]

---

[1] Ruxin J, et al. 2005. Emerging consensus in HIV/AIDS, malaria, tuberculosis, and access to essential medicines. *Lancet* 365(9459):618-21.
[2] Bigdeli M, Jacobs B, Tomson G, Laing R, Ghaffar A, Dujardin B, et al. Access to medicines from a health system perspective. *Health Policy and Planning* 2012;1–13. doi:10.1093/heapol/czs108.
[3] World Health Organization. 2004. Equitable access to essential medicines: a framework for collective action. Policy Perspectives on Medicines 8: 1–6.
[4] Frost LJ, Reich MR. 2010. How do good health technologies get to poor people in poor countries. Boston, MA: Harvard Center for Population and Development Studies.

Existing barriers prevent many women and children from accessing simple, lifesaving treatments and interventions in low- and middle- income countries. The pharmaceutical management framework (Figure 2) provides the underpinning for improving access to medicines as described above. Pharmaceutical management involves four basic functions: selection, procurement, distribution, and use.

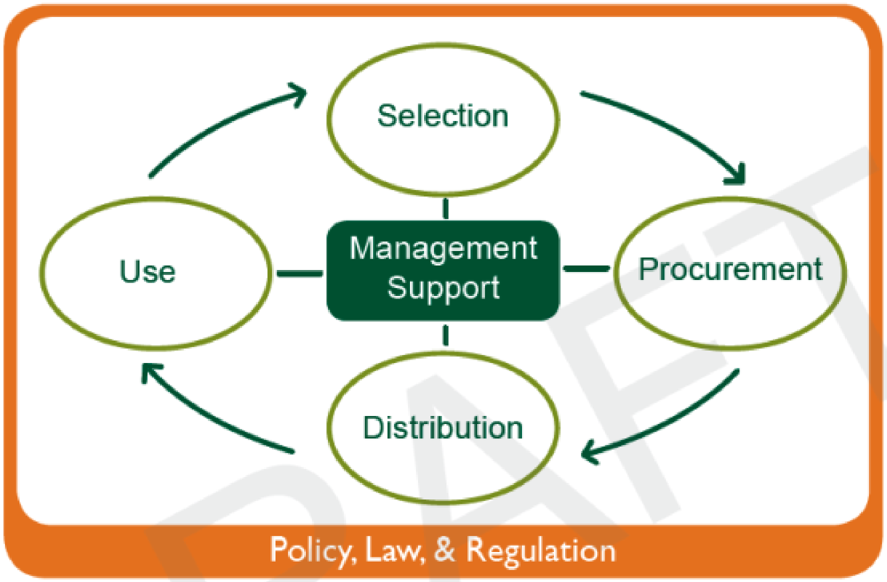

Figure 2. Pharmaceutical management framework. Each major function builds on the previous function and leads logically to the next A breakdown in one part of the framework leads to failure of the whole pharmaceutical management process.[5]

Selection involves reviewing the prevalent health problems, identifying treatments, choosing individual medicines and dosage forms, and deciding which medicines will be available at each level of health care. **Procurement** includes quantifying medicine requirements, selecting procurement methods, and assuring pharmaceutical quality. **Distribution** includes inventory management, stores management, and delivery to drug depots and health facilities. **Use** includes diagnosing, prescribing, dispensing, and proper consumption by the patient. The entire framework relies on policies, laws, and regulations, which when supported by good governance, support the public commitment to essential medicine supply. At the center is a core of management support systems: organization, financing and sustainability, information management, and human resources management. When the separate tasks are performed independently and disjointedly and not as part of a system, costs rise, shortages become common, and patients suffer.

[5] Ibid.

DETERMINANTS OF ACCESS TO LIFESAVING COMMODITIES

While the following determinants are not the only policy and pharmaceutical system factors that determine access, they are critical for the reasons outlined below.

Policy

The World Health Organization (WHO) defines essential medicines as those that satisfy the needs of the majority of the population and therefore should be available at all times. The rationale for the selection and use of a limited number of essential medicines is that it improves the supply of medicines and rational prescribing and lowers costs; in fact, the appropriate use of essential medicines is one of the most cost-effective strategies a country can enact. By 2007, 86 percent of countries had national essential medicines lists (EMLs), of which at least 69% had been updated in the previous five years.[6] Figure 3 shows how the essential medicines concept applies to different levels of the health care system.

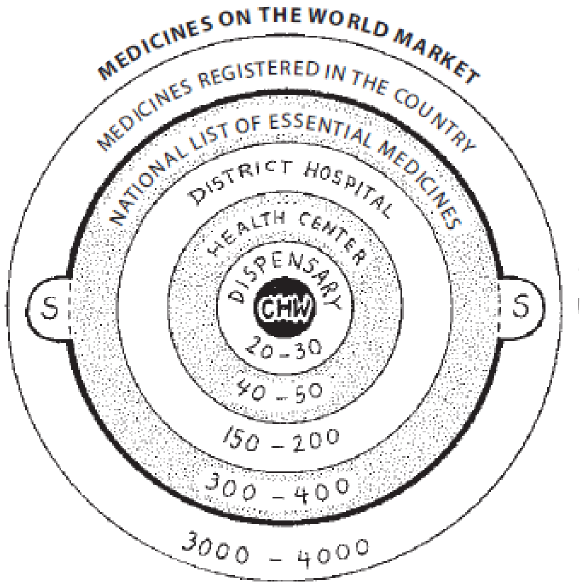

Notes: S = specialist medicines available to special hospitals and departments in the public sector; CHW = community health worker, who typically has an essential medicines list of 12 to 18 items.

Figure 3. Selection of essential medicines by level of health care

For public-sector supply programs, policies that focus on a limited number of essential medicines simplifies procurement and logistics efforts by reducing the number of products that must be purchased, distributed, and monitored. Often the EML is used as a procurement list for the public sector, so if a product is not on it, it will not be procured. In addition, because essential medicines are usually available from multiple suppliers, competition reduces prices. National essential medicine policies are also significantly associated with better quality use of medicines in low-income countries.[7] Because EMLs are viewed as the cornerstone to the essential medicines policy— for example, the piece that drives expenditure priorities—whether a country has a national EML and which products it contains is used as a proxy for the health of the public pharmaceutical supply chain.[8,9] Essential medicines are significantly better stocked than nonessential medicines, and a product's inclusion on the national EML

[6] Management Sciences for Health. 2012. *MDS-3: Managing Access to Medicines and Health Technologies*. Arlington, VA: Management Sciences for Health.
[7] Holloway KA, Henry D. 2014. WHO essential medicines policies and use in developing and transitional countries: an analysis of reported policy implementation and medicines use surveys. *PLoS Med* 11(9): e1001724. doi:10.1371/journal.pmed.1001724.
[8] Ruxin J, Paluzzi JE, Wilson PA, Tozan Yi, Kruk M, Teklehaimanot A. 2005. Emerging consensus in HIV/AIDS, malaria, tuberculosis, and access to essential medicines. *Lancet*. 12-18; 365(9459):618-21.
[9] Hill S, Yang A, Bero L . 2012. Priority medicines for maternal and child health: a global survey of national essential medicines lists. *PLoS ONE* 7(5): e38055. doi:10.1371/journal.pone.0038055.

is the first step toward assuring availability, however, other barriers can block access to priority medicines.[10,11] For example, Uganda updated its essential medicines and supplies list in 2012 and classified each product by whether it was vital, essential, or necessary, which gives procurement priority when resources are limited; however, because child-friendly formulations were not emphasized in the list as priority procurements, an assessment suggested that this contributed to lack of availability and use of recommended products.[12]

Standard treatment guidelines (STGs) are systematically developed documents recommending appropriate treatments for specific clinical problems. These guidelines usually reflect the consensus on the optimal treatment options within a health facility or health system. The STGs and EML should be linked; in other words, a recommended treatment should also be included on the EML. The information emphasizes the country's common diseases and complaints and the various treatment alternatives. Information on medicines in STGs is usually limited to strength, dosage, and duration. Most guidelines indicate a treatment of first choice.

An extensive analysis of how essential medicine policies affect medicine use in developing and transitional countries found that undergraduate training for doctors and nurses in STGs had the greatest impact on the quality of medicines use.[13] STGs are an important tool to facilitate rational use of medicines and therefore the quality of services, which is a crucial component of access. A well-implemented essential medicines program with updated EML and STGs can have a strong impact on a country's health services.
Some examples of indicators to measure policy factors in access to medicines are:

- Percent of countries with a current EML (updated in the last 2 years)
- Percent of countries with an EML that includes all and specific tracer RMNCH medicines
- Percent of countries with all and specific RMNCH tracer medicines in STGs
- Percent of countries where the all and specific RMNCH tracer medicines in the STG are also on the EML
- Percent of countries where national policy includes community management of diarrhea and pneumonia

Regulatory

To protect public health, a country's pharmaceutical regulations need to address the manufacture, trade/sales, and use of medicines, including product quality assurance. If a drug product does not meet established quality standards, passes its expiration date, or has been degraded by storage conditions, the consequences can include treatment failure, adverse reactions, or even death. A country's pharmaceutical quality assurance system includes technical and managerial activities such as evaluating pharmaceutical product documentation, performing or reviewing quality-

---

[10] Bazargani YT, Ewen M, de Boer A, Leufkens HGM, Mantel-Teeuwisse AK. 2014. Essential medicines are more available than other medicines around the globe. *PLoS ONE* 9(2): e87576. doi:10.1371/journal.pone.0087576.

[11] Hill S, Yang A, Bero L. 2012. Priority medicines for maternal and child health: a global survey of national essential medicines lists. *PLoS ONE* 7(5): e38055. doi:10.1371/journal.pone.0038055.

[12] Nsabagasani X, Ogwal-Okeng J, Mbonye A, Ssengooba F, Muhumuza S, Holme Hansen E. Availability and utilization of the WHO recommended priority lifesaving medicines for under five-year old children in public health facilities in Uganda: a cross-sectional survey. *J Pharm Policy Pract.* 2015; 8(1): 18.

[13] Holloway KA, Henry D. 2014. WHO essential medicines policies and use in developing and transitional countries: an analysis of reported policy implementation and medicines use surveys. *PLoS Med* 11(9): e1001724. doi:10.1371/journal.pmed.1001724.

control laboratory tests, selecting reliable suppliers, and conducting inspections throughout the distribution network (Figure 4).

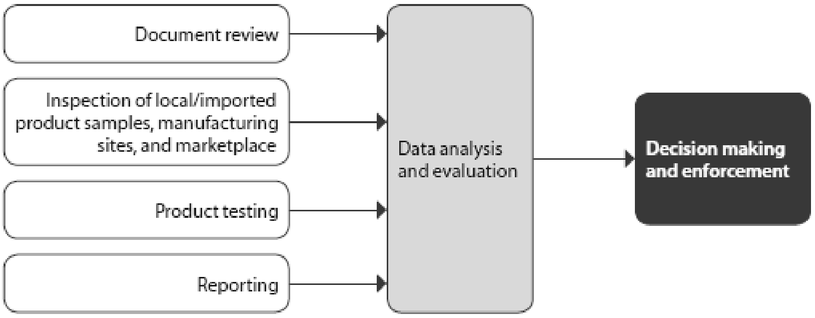

Figure 4. Pharmaceutical quality assurance framework

One of the major responsibilities of a country's drug regulatory authority is to maintain a registration system to ensure that pharmaceutical products approved for sale and distribution are efficacious, safe, and manufactured according to official standards. An inefficient or poorly functioning registration system can prevent lifesaving products from being available; for example, as the health authorities in Namibia were scaling up antiretroviral therapy, a backlog of about 1,000 product registration dossiers held up the authorization of important fixed-dose and pediatric antiretrovirals.[14]

Some examples of indicators to measure regulatory factors in access to RMNCH medicines are:

- Percentage of countries where all tracer RMNCH medicines have at least one product registered for use in country
- Percentage of countries where quality problems are reported
- Percentage of countries where medicines (including RMNCH medicines) products are routinely sampled for quality testing

Procurement

In most resource-limited countries, pharmaceutical purchases represent the single largest health expenditure after human resources. An effective procurement process should ensure the availability of the right medicines in the right quantities, at reasonable prices, and at recognized standards of quality. Given the impact of procurement activities on the operation and effectiveness of health services, these activities must be carried out by skilled staff who have access to reliable inventory and consumption information.

When a health system's procurement program is centrally managed, it functions as a type of pooled procurement system for health regions, districts, and individual facilities. Centralized procurement allows for economies of scale, which helps reduce prices and improve market presence, which leads to better supply security. For example, after China introduced a national EML that included central procurement through competitive bidding by provincial authorities,

[14] Pereko D, et al. Improving the availability of ARVs in Namibia by using policy change to streamline the drug registration process. Presented at the XVI International AIDS Society Conference, 13–18 August 2006, Toronto.

inpatient drug expenditures fell by 26% and total expenditures per admission fell by 10%.[15] By increasing procurement efficiency through centralized procurement, the price of essential medicines in China fell by 16.9% between 2009 and 2011.[16]

An example of an indicator to measure procurement issues in access to medicines is the percentage of countries where RMNCH commodities are procured centrally.

Financing

Having RMNCH policies and implementation plans in place is important, but without complementary political will and funding, plans are ineffectual. For example, Tanzania has experienced remarkable improvements in decreasing child mortality and has met its MDG 4, but maternal and neonatal mortality have lagged behind, even though interventions for these populations were included in Tanzania's One Plan to address maternal and neonatal health. Investigating the disparity, a recent Countdown analysis cited an inequitably financed and implemented plan that strongly prioritized child health services over maternal and newborn health interventions.[17]

Based on research showing that user fees can easily cause a decrease in the utilization of health care services, most international agencies discourage users' fees for preventive and some curative care, including the World Bank and World Health Organization.[18] Although user fees can contribute to budgets for services and pharmaceuticals that would otherwise not be available, evidence shows that fees are usually a major detriment to access to the community's poorest people, because exemption plans are often nonfunctional.[19] A global analysis of how essential medicines policies affect medicine use found that policies to dispense essential medicines free of charge to all patients and to children under five had one of the largest positive effects.[20]

Some examples of indicators to measure financing issues in access to medicines are:

- % of countries with a costed MNCH plan
- % of countries with fees for services in the public sector
- % of countries with fees for services where women and children under 5 are exempt from paying for RMNCH services or medicines
- % of countries where the RMNCH commodities are provided free of charge in public sector

---

[15] Zhang X, Wu Q, Liu G, et al. 2014. The effect of the National Essential Medicines Policy on health expenditures and service delivery in Chinese township health centres: evidence from a longitudinal study. *BMJ Open*;4:e006471. doi:10.1136/bmjopen-2014- 006471.

[16] Ibid.

[17] Afnan-Holmes H, Magoma M, John T, Levira F, Msemo G, Armstrong CE, et al. 2015. Tanzania's Countdown to 2015: an analysis of two decades of progress and gaps for reproductive, maternal, newborn, and child health, to inform priorities for post-2015. *Lancet Glob Health* 3: e396–409.

[18] Liu X, O'Dougherty S. 2005. Paying for public health services: financing and utilization. In: Spending Wisely: Buying Health Services for the Poor. AS Preker and JC Langenbrunner (eds.). Washington, D.C.: World Bank.

[19] Gottret, P. and G. Schieber. 2006. Health Financing Revisited. Washington, D.C.: World Bank.

[20] Holloway KA, Henry D. 2014. WHO essential medicines policies and use in developing and transitional countries: an analysis of reported policy implementation and medicines use surveys. *PLoS Med* 11(9): e1001724. doi:10.1371/journal.pmed.1001724.

Supply chain management

A country's medicines distribution can be based on a "pull" system, where health facilities determine types and quantities of products they need and place orders with a supplier or a "push" system, where a supplier sends health facilities a predetermined selection and quantity of products at a scheduled time (also known as a kit system). In Uganda, the National Medical Store re-instituted a kit system for the two lowest levels of health care, which had been experiencing chronic stock-outs. The content and quantities for the two separate kits were customized to the level of care. However, each health center receives its customized kit six times a year regardless of the center's catchment area population or patient load. A 2013 evaluation of the system showed that most pharmaceuticals and supplies were either under or oversupplied.[21] Only about 20% of items appeared to be adequately supplied. As a result, the government is now moving back toward having the lower level facilities place their own orders. Other countries, such as Nepal, have reduced stock-outs when they shifted to a pull system. Although a pull system is the gold standard, it requires adequately skilled staff and information management.[22,23,24]

While this indicator on pull distribution methods is not the only one to assess supply chain function, it is relatively easy to measure to supply a system snapshot. Many things cause stock-outs at the central medical stores and can indicate problems in quantification, procurement, or funding. Central stock-outs also predict stock-outs further down the supply chain, which reduce availability at the service level. An informed push model, which uses trained logisticians to monitor consumption and supply at multiple facilities has been successful, but not implemented in many countries.[25]

Some examples of indicators to measure supply chain management at the central level are:

- % of countries with a pull (demand-based) distribution method to health facilities
- % of countries with stock outs of RMNCH tracer products reported at the central medical store in last 3 years

Other supply chain management indicators are used as well, but these are readily available in existing data sets, such as the RMCNH landscape synthesis and the WHO regulatory and procurement survey.

Information systems

Accurate and timely information on a variety of pharmaceutical system parameters affects the ability to conduct accurate quantification, plan procurement, budget, and mobilize resources. A good logistics management information system (LMIS) is critical to being able to quantify how much of a product should be procured and what the stock status is at all levels of the health system to assure access to lifesaving commodities. In Uganda, for example, the national LMIS

---

[21] Ministry of Health Uganda, Pharmacy Division. Assessment of the first region-specific essential medicines kit supply system in Uganda: 2013. Kampala: Ministry of Health.

[22] Kit System Management. In: Management Sciences for Health. 2012. MDS-3: Managing Access to Medicines and Health Technologies. Arlington, VA: Management Sciences for Health.

[23] Nepal Family Health Program Technical Brief #13. Undated. Available at: http://nfhp.jsi.com/Res/Docs/techbrief13-pull_sys.pdf.

[24] Mikkelsen-Lopez I, Cowley P, Kasale H, Mbuya C, Reid G, de Savigny D. Essential medicines in Tanzania: does the new delivery system improve supply and accountability? *Health Systems* (2014) 3, 74–81.

[25] Daff et al 2014 *Glob Health Sci Pract*. 2014;2(2):245-252.

now includes stock information that is easily available to the government's procurement planning unit, which monitors and reports on the medicine and commodity stock status at all levels of the health system, from the lowest level health facilities to the central medical store. Thanks to this, in one year, the unit was able to initiate warehouse transfers of HIV commodities worth approximately US$6.8 million, which prevented product stock-outs and disruption of health services downstream.[26]

Some examples of indicators of information systems include:

- Percentage of countries with an LMIS to track stock-level/consumption of medicines (paper-based, electronic, or mobile)
- Percentage of countries where all tracer RMNCH commodities are included in existing LMIS

CONCLUSIONS

Countries need indicators on policy and system factors that influence access to RMNCH medicines that they can monitor easily at the national level to ensure progress to SDG 3. The examples mentioned here are included already in some data collection efforts and can be relatively easily included in national level monitoring activities.

[26] Securing Ugandans' Right to Essential Medicines Program. Final Report: 2009-2014. Arlington, VA: Management Sciences for Health.
