# Supplementary material for: How to assure access of essential RMNCH medicines by looking at policy and systems factors: an analysis of countdown to 2015 countries
Source: BMC Health Serv Res. 2018 Dec 7;18:952. doi: 10.1186/s12913-018-3766-6 (PMC6286577; doi:10.1186/s12913-018-3766-6)
Supplement: Supplementary file 2 — Indicators and data sources. (PDF 70 kb) [file 12913_2018_3766_MOESM2_ESM.pdf]

Additional File 2: Indicators and Data Sources

Indicators	Data Sources
**Policy**	
1. % of countries with a current (updated in the last 2 years) essential medicines list (EML)	WHO Essential Medicines and Health Products Information Portal for national EMLs
2. % of countries with an EML that includes tracer RMNCH medicines *	WHO health systems and policy survey 2014 dataset<br>and<br>WHO Regulatory and procurement survey 2014 complemented by RMNCH Landscape Synthesis for certain countries depending on commodity as RH commodities were not included in WHO health systems and policy survey 2014 dataset
3. % of countries with RMNCH tracer medicines in standard treatment guidelines (STGs)	WHO health systems and policy survey 2014 dataset<br>WHO Regulatory and procurement survey 2014
4. % of countries where RMNCH tracer medicines in the STG are also on the EML*	WHO health systems and policy survey 2014 dataset<br>WHO Regulatory and procurement survey 2014<br>RMNCH Landscape Synthesis
5. % of countries with a national policy on the use of community management of pneumonia	WHO health systems and policy survey 2014 dataset
6. % of countries with a national policy on the use of community management of diarrhea	WHO health systems and policy survey 2014 dataset
**Regulatory**	
7. % of countries where tracer medicines have at least one product registered for use in country *	WHO Regulatory and procurement survey 2014<br>RMNCH Landscape Synthesis
8. % of countries where quality problems are reported	WHO Regulatory and procurement survey 2014<br>RMNCH Landscape Synthesis
9. % of countries where medicines (including RMNCH medicines) products are routinely sampled for quality testing	WHO Regulatory and procurement survey 2014<br>RMNCH Landscape Synthesis
**Procurement**	
10. % of countries where RMNCH commodities are procured centrally *	WHO Regulatory and procurement survey 2014<br>RMNCH Landscape Synthesis
**Financing**	
11. % of countries with a costed MNCH plan	WHO health systems and policy survey 2014 dataset
12. % of countries with fees for services in the public sector	WHO health systems and policy survey 2014 dataset
13. % of countries with fees for services where women and children under 5 are exempt from paying for RMNCH services or medicines	WHO health systems and policy survey 2014 dataset
14. % of countries where the RMNCH commodities are provided free of charge in public sector *	WHO Regulatory and procurement survey 2014
**Supply Chain Management**	
15. % of countries with a pull (demand-based) distribution method to health facilities	WHO Regulatory and procurement survey 2014

16. % of countries with stock outs of RMNCH tracer products reported at CMS in last 3 years *	WHO Regulatory and procurement survey 2014
**Information systems**	
17. % of countries with a LMIS system to track stock-level/consumption of medicines (paper, electronic, or mobile)	WHO Regulatory and procurement survey 2014
18. % of countries where all tracer RMNCH commodities are included in existing LMIS*	WHO Regulatory and procurement survey 2014<br>RMNCH Landscape Synthesis
