# Supplementary material for: How to assure access of essential RMNCH medicines by looking at policy and systems factors: an analysis of countdown to 2015 countries
Source: BMC Health Serv Res. 2018 Dec 7;18:952. doi: 10.1186/s12913-018-3766-6 (PMC6286577; doi:10.1186/s12913-018-3766-6)
Supplement: Supplementary file 3 — Reproductive and maternal health commodity policy and systems indicators for a subset of countries (countries arranged in order of increasing gap with CD 2015 targets). (PDF 94 kb) [file 12913_2018_3766_MOESM3_ESM.pdf]

Additional file 3: Reproductive and maternal health commodity policy and systems indicators for a subset of countries (countries arranged in order of increasing gap with CD 2015 targets)

	Country A	Country B	Country C	Country D	Country E	Country F	Country G	Country H	Country I	Country J
**POLICY**										
Policy for AMTSL	Y	Y	Y	Y	Y	Y	Y	Y	Y	Y
All RH commodities on EML (3)	N 2/3	N 0/3	N 2/3	N 2/3	N 1/3	N 1/3	Y	Y	N 1/3	Y
All MH commodities on EML (4)	Y	Y	N 3/4	Y	Y	N 3/4	Y	Y	Y	Y
EML is current	N	Y	Y	N	N	N	N	Y	N	N
All RH commodities on STG (3)	Y	N 1/2	Y 2/2	Y 2/2	N 1/2	Y	Y	Y	Y 1/1	Y
All MH commodities on STG (4)	Y	N 3/4	Y	Y	Y	Y 3/3	Y 3/3	Y	Y	Y
**REGULATORY**										
At least 1 product registered for all RH commodities	Y	-	Y	N 2/3	N 2/3	N 2/3	N 0/3	N 2/3	-	N 2/3
At least 1 product registered for all MH commodities	Y	-	N 2/4	Y	N 3/4	N 3/4	N 3/4	Y	-	N 3/4
Quality problems reported	Y	-	Y	Y	Y	Y	-	Y	-	Y
Medicines sampled for quality testing	Y	-	Y	Y	Y	Y	-	Y	-	Y
**PROCUREMENT**										
All RH products procured centrally in last year (3)	N 2/3	N 1/3	N 1/3	Y	N 2/3	N 0/3	Y	Y	N 1/3	Y
All MH products procured centrally in last year (4)	Y	Y	N 3/4	Y	Y	Y 3/3	Y 3/3	Y	N 2/4	Y
**FINANCING**										
All RH commodities provided free of charge (3)	Y	Y 1/1	Y 2/2	Y 2/2	Y 2/2	Y	N 1/3	N 1/3	Y 1/1	Y
All MH commodities provided free of charge (4)	Y	Y	Y	Y	Y 3/3	Y 3/3	Y	N 0/4	N 0/3	Y
Costed MH plan	Y	Y	Y	Y	Y	Y	-	Y	Y	Y
Fees for services in public sector	N	N	Y	N	N	Y	Y	Y	Y	Y
Women exempt	-	-	Y	-	-	Y	-	N	N	N
**SUPPLY CHAIN MANAGEMENT**										
Pull distribution method	Y	N	Y	N	-	N	N	N	-	N
No Stock outs at CMS in last 3 years for RH	1/3	-	*	*	2/2	2/3	*	*	1/1	0/3
No Stock outs at CMS in last 3 years for MH	¾	-	¼	¾	¾	3/3	1/3	*	*	0/1
**INFORMATION SYSTEMS**										
LMIS system to track medicines	Y	N	Y	Y	Y	Y	Y	-	Y	Y
All RH commodities tracked by LMIS (3)	Y	N 0/3	Y	N 1/3	Y 2/2	Y	Y	Y 2/2	N 1/3	Y
All MH commodities tracked by LMIS (4)	Y	N 0/4	N 3/4	N 0/4	Y	Y 3/3	Y 3/3	Y	N 2/4	Y
**HEALTH OUTCOME**										
MMR from Countdown report 2015	420	400	410	360	510	1100	650	320	450	470
CD 2015 MMR target	350	300	230	200	280	580	280	130	170	130
pending gap as % of 2014 MMR	17	25	44	44	45	47	57	59	62	72

Key: Colors depend on # of commodities fulfilling indicator condition for each country. Green: > 75% of the commodities for that indicator; yellow 60-75% of the commodities; red: <60% of the commodities. Where information was available for the full set of commodities there is a * in the cell; where information was available for less than the full number of commodities the number was specified. Where there was no data the cell was left white. Health outcome: mortality gap was shaded red if over 60% and yellow if between 25 and 60%
