# Supplementary material for: How to assure access of essential RMNCH medicines by looking at policy and systems factors: an analysis of countdown to 2015 countries
Source: BMC Health Serv Res. 2018 Dec 7;18:952. doi: 10.1186/s12913-018-3766-6 (PMC6286577; doi:10.1186/s12913-018-3766-6)
Supplement: Supplementary file 4 — Newborn and child health commodity policy and systems indicators for a subset of countries (countries arranged in order of increasing gap with CD 2015 targets). (PDF 97 kb) [file 12913_2018_3766_MOESM4_ESM.pdf]

Additional file 4: Newborn and child health commodity policy and systems indicators for a subset of countries (countries in order of increasing gap with CD 2015 targets)

	Country E	Country A	Country D	Country C	Country H	Country G	Country F	Country B	Country I	Country J
**POLICY**										
Policy for community management of child	Y	Y	Y	N	Y	Y	Y	Y	Y	N
All NH commodities on EML (5)	Y	N 4/5	N 4/5	N 4/5	Y	Y	N 4/5	Y	N 4/5	Y
All CH commodities on EML (3)	N 2/3	N 2/3	Y	Y	Y	Y	N 2/3	Y	N 2/3	Y
EML is current	N	N	N	Y	Y	Y	N	Y	N	N
All NH commodities on STG (5)	N 4/5	Y 4/4	Y	Y 3/3	Y 3/3	Y	Y	N 3/5	Y 4/4	Y
All CH commodities on STG (3)	Y	Y	Y	Y	Y 2/2	Y	Y	Y	Y 2/2	Y
**REGULATORY**										
At least 1 product registered for all NH	N 4/5	N 4/5	N 4/5	N 2/5	N 4/5	Y	N 3/5	-	-	Y
At least 1 product registered for all CH	Y	N 1/3	Y	N 2/3	N 2/3	N 2/3	Y	-	-	Y
Quality problems reported	Y	Y	Y	Y	Y	-	Y	-	-	Y
Medicines sampled for quality testing	Y	Y	Y	Y	Y	-	Y	-	-	Y
**PROCUREMENT**										
All NH products procured centrally in last year (5)	Y	Y 4/4	Y 3/3	Y 4/4	Y	Y	Y	N 4/5	N 4/5	N 4/5
All CH products procured centrally in last year (3)	Y	N 2/3	N 0/2	Y	Y	Y	Y	Y	N 1/3	Y
**FINANCING**										
All NH commodities provided free of charge (5)	Y 4/4	Y 4/4	Y	-	N 0/3	N 0/5	Y	N 4/5	N 0/4	Y
All CH commodities provided free of charge (3)	Y 2/2	Y	Y	Y	N 0/2	N 0/3	Y	Y	N 1/2	Y
Costed NH plan	Y	Y	Y	Y	Y	-	Y	Y	Y	Y
Costed CH plan	Y	Y	Y	Y	Y	-	Y	Y	Y	Y
Fees for services in public sector	N	N	N	Y	Y	Y	Y	N	Y	Y
Newborns exempt	-	-	-	Y	N	N	Y	-	N	N
Children exempt	-	-	-	Y	N	N	Y	-	N	N
**SUPPLY CHAIN MANAGEMENT**										
Pull distribution method	-	Y	N	Y	N	N	N	N	-	N
No Stock outs at CMS in last 3 years for NH	*	4/4	4/5	2/4	*	0/5	1/1	-	4/4	-
No Stock outs at CMS in last 3 years for CH	2/3	1/2	1/2	*	*	0/3	-	-	2/2	0/1
**INFORMATION SYSTEMS**										
LMIS system to track medicines	Y	Y	Y	Y	-	Y	Y	N	Y	Y
All NH commodities tracked by LMIS (5)	Y	Y 4/4	N 0/3	Y 3/3	Y	Y	Y	N	Y 4/4	Y 4/4
All CH commodities tracked by LMIS (3)	Y	Y 2/2	N 0/3	Y	Y 2/2	Y	Y	N 0/3	Y 1/1	Y
**HEALTH OUTCOME**										
U5 MR from Countdown report 2015	64	59	55	49	47	94	120	91	78	71
CD 2015 U5MR target	81	68	62	55	47	79	88	60	49	25
Pending gap as % of 2014 U5MR	-27	-15	-13	-12	0	16	27	34	37	65

Key: Same country codes as for additional file 3. Colors depend on # of commodities fulfilling indicator condition for each country. Green: > 75% (i.e. 3/4 or 4/5 of the commodities for that indicator; yellow 60-75% of the commodities; red: <60% (or 2/3) of the commodities. Where information was available for the full set of commodities there is a * in the cell; where information was available for less than the full number of commodities the number was specified. Where there was no data the cell was left white. Health outcome: mortality gap was shaded red if over 60% and yellow if between 25 and 60% and green for no gap or negative gap
